# Feeding the gut-immune axis: dietary, prebiotic, and probiotic strategies to target persistent inflammation in ART-treated HIV: a narrative review

**DOI:** 10.3389/fimmu.2026.1877822

**Published:** 2026-07-17

**Authors:** Aaron A. Fletcher, Ziad Koberssy, Joviane Daher, Nicolas Moussallem, Grace A. McComsey

**Affiliations:** 1 School of Medicine, Case Western Reserve University, Cleveland, OH, United States; 2 Clinical Research Center, University Hospitals, Cleveland Medical Center, Cleveland, OH, United States

**Keywords:** *Akkermansia muciniphila*, fecal microbiota transplantation, gut microbiome, HIV, inflammation, Mediterranean diet, microbial translocation, NF-κB

## Abstract

People living with HIV (PWH) experience ongoing systemic inflammation driven by gut dysbiosis, epithelial barrier disruption, and microbial translocation, despite antiretroviral therapy (ART). This review examines evidence from randomized controlled trials, mechanistic studies, systematic reviews, and meta-analyses evaluating nutritional and microbiome-based interventions to reduce inflammation in PWH. Reduced production of short-chain fatty acids (SCFAs) by the gut microbiota has been observed to precede morbidity and mortality in PWH, with SCFAs, mainly butyrate, exerting immunomodulatory effects through promoting regulatory T-cell differentiation via histone deacetylase inhibition and G protein-coupled receptor 43 (GPR43) and GPR109A signaling, suppressing nuclear factor kappa B (NF-κB)-mediated pro-inflammatory cytokine production, and enhancing epithelial tight junction integrity. Probiotics, prebiotics, synbiotics, and fecal microbiota transplantation have demonstrated reductions in inflammatory biomarkers including soluble CD14 (sCD14), lipopolysaccharide-binding protein (LBP), and high-sensitivity C-reactive protein (hsCRP). The Mediterranean diet, omega-3 fatty acids, and polyphenol-rich foods represent an underexplored area as modulators of gut microbiota composition and SCFA production. Existing gaps in the literature include a lack of trials with clinically meaningful endpoints, optimal probiotic strains and doses, and lack of randomized trials evaluating anti-inflammatory dietary patterns in PWH. We propose a research agenda prioritizing Mediterranean diet intervention trials, precision microbiome interventions, and combination approaches integrating dietary modification with microbiome-targeted therapies. Lastly, we provide practical nutritional recommendations for clinicians managing PWH.

## 1 Introduction

The population of people living with human immunodeficiency virus (HIV) receiving antiretroviral therapy (ART) continues to expand globally, with projections estimating over 900, 000 individuals on treatment in the United States alone by 2030 (1). Despite sustained viral suppression, people living with HIV (PWH) continue to experience a disproportionate burden of non-acquired immunodeficiency syndrome (AIDS) comorbidities including cardiovascular disease, malignancy, metabolic syndrome, and neurocognitive impairment that drive excess morbidity and mortality compared with the general population (1, 2). Modeling studies forecast that multimorbidity among ART-treated PWH will rise from 63% in 2020 to 70% by 2030 (1). This persistent health disparity has been attributed to HIV-associated premature aging with multimorbidity emerging roughly a decade earlier in PWH compared to individuals without HIV (2).

Central to this phenomenon is chronic, low-grade systemic inflammation that persists despite effective antiretroviral therapy and virologic suppression. Throughout this review, persistent inflammation refers to ongoing immune activation characterized by sustained elevations in inflammatory biomarkers. Elevated levels of biomarkers including high-sensitivity C-reactive protein (hsCRP), interleukin-6 (IL-6), and soluble cluster of differentiation 14 (sCD14) often remain present in virologically suppressed PWH and have been associated with cardiovascular events, non-AIDS morbidity, and mortality (3, 4). Further, expansion of cluster of differentiation 8 (CD8) T-cell subsets and a lower cluster of differentiation 4 (CD4)/CD8 ratio have been linked to persistent immune dysfunction, multimorbidity, and non-AIDS illnesses (5). The mechanisms underlying this residual inflammation are multifactorial, but converging evidence implicates the gut-immune axis as a central mediator. HIV infection inflicts profound and early damage to the gastrointestinal tract, resulting in a significant reduction of gut-resident CD4+ T cells, specifically T helper 17 (Th17) and T helper 22 (Th22) cells essential for mucosal barrier integrity, early during infection (6). This results in a cascade of pathological changes, including epithelial barrier disruption, increased intestinal permeability, and translocation of microbial products (including lipopolysaccharide [LPS], bacterial deoxyribonucleic acid (DNA), and the fungal β-D-glucan) from the gut lumen into systemic circulation (7–9). Microbial translocation, in turn, promotes chronic immune activation (6, 7).

Accompanying these structural and immunological changes is HIV-associated gut dysbiosis. Multiple studies have reported an increased abundance of inflammation-associated taxa (Proteobacteria, Enterobacteriaceae, Erysipelotrichaceae) alongside depletion of obligate anaerobic and potentially beneficial taxa, including Clostridia, Ruminococcaceae, *Anaerostipes*, Rikenellaceae, and *Alistipes* (7, 10). These dysbiotic signatures persist despite sustained viral suppression and are associated with markers of microbial translocation (sCD14, LPS) and systemic inflammation (tumor necrosis factor-α [TNF-α], interleukin-1β [IL-1β]) (7, 10, 11). The bacterium *Akkermansia muciniphila*, commonly considered a marker of gut barrier integrity, is reduced in PWH and has emerged as a potential therapeutic target (12). The functional consequences of HIV-associated dysbiosis extend beyond taxonomic shifts to encompass impaired production of short-chain fatty acids (SCFAs), including butyrate, propionate, and acetate, the principal metabolites produced through bacterial fermentation of dietary fiber (13–15). SCFAs exert pleiotropic immunomodulatory effects through several mechanisms, including activation of G protein-coupled receptors (GPR43, GPR41, GPR109A) expressed on epithelial and immune cells, inhibition of histone deacetylases (HDACs) with downstream effects on gene transcription, and direct metabolic reprogramming of immune cell function (13, 15, 16). Butyrate serves as the primary energy source for colonocytes and is essential for maintaining epithelial tight junction integrity, thereby limiting microbial translocation (13). Beyond barrier maintenance, these mechanisms collectively promote anti-inflammatory immune responses, including enhanced regulatory T-cell differentiation, suppression of NF-κB-mediated pro-inflammatory cytokine production, modulation of macrophage and dendritic cell function toward tolerogenic phenotypes, and support of immunoglobulin A (IgA)-producing plasma cells critical for mucosal defense (13, 15–18).

A 2023 study found that impaired gut microbiota-mediated SCFA production precedes morbidity and mortality in ART-treated PWH (14). Using stool and serum samples obtained prior to the onset of comorbidities, the investigators demonstrated that HIV-associated microbiome alterations, particularly impaired microbial conversion of lactate to propionate, preceded mortality, thereby establishing a temporal link between SCFA deficiency and adverse clinical outcomes. Notably, serum SCFA concentrations, rather than fecal levels, more accurately reflected the capacity of the gut microbiota to produce SCFAs and were inversely associated with inflammatory markers, including IL-6, kynurenine-to-tryptophan ratio, soluble urokinase plasminogen activator receptor, and sCD14. These results position SCFAs as a promising predictive biomarker and potential mechanistic target for future intervention.

Given the pivotal role of the gut-immune axis in HIV-associated inflammation, strategies to restore gut microbial ecology, enhance SCFA production, and reinforce epithelial barrier integrity represent a promising adjunctive approach to effective ART. Probiotic, prebiotic, and synbiotic interventions have produced variable but promising effects on inflammatory biomarkers in PWH, including reductions in sCD14, lipopolysaccharide-binding protein (LBP), hsCRP, and IL-6 (19–22). Fecal microbiota transplantation (FMT) has shown capacity to modulate the inflammatory proteome in PWH, identifying bacterial drivers of inflammation (23), while earlier work demonstrated safe engraftment and reduced intestinal damage markers (24). Emerging evidence suggests that dietary interventions, particularly Mediterranean-style diets rich in fiber, polyphenols, and omega-3 fatty acids, may favorably modulate gut microbiota composition and inflammatory pathways, though randomized evidence in PWH remains limited (25–27).

**Figure 1:**
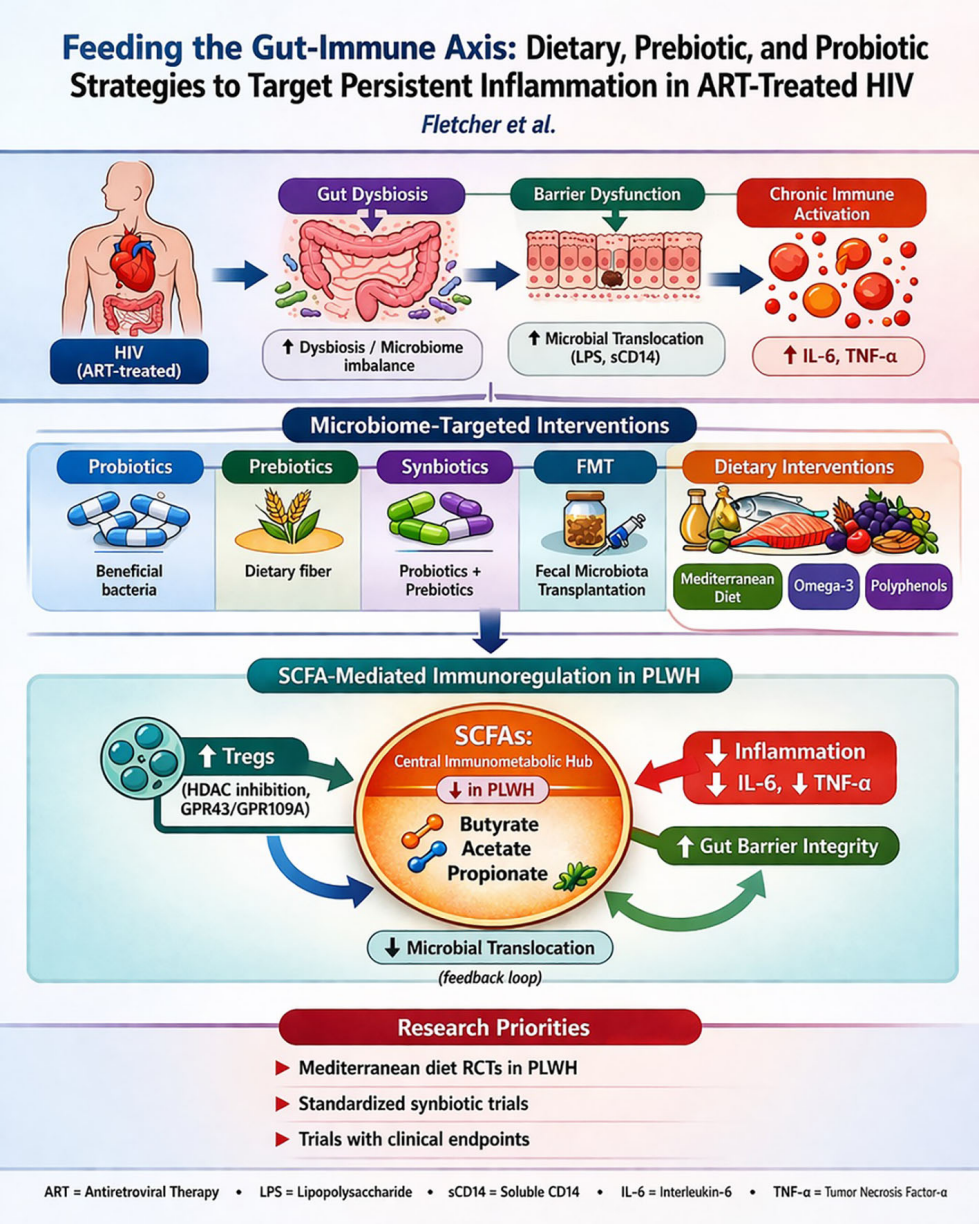
Pathways linking gut dysbiosis, microbial translocation, and persistent inflammation in antiretroviral therapy-treated HIV. Gut dysbiosis and impaired intestinal barrier integrity promote microbial translocation and chronic immune activation. Dietary and microbiome-targeted interventions, including probiotics, prebiotics, synbiotics, fecal microbiota transplantation, and dietary modification, influence short-chain fatty acid production, regulatory T-cell activity, gut barrier integrity, microbial translocation, and inflammatory signaling.

This review synthesizes current evidence on nutritional and microbiome-based interventions to reduce inflammation in ART-treated PWH, with emphasis on the immunological mechanisms underlying therapeutic effects. We integrate findings from randomized controlled trials (RCTs) of probiotics, prebiotics, synbiotics, and FMT with mechanistic studies of SCFA-mediated immune regulation. We evaluate the evolving evidence for dietary strategies including the Mediterranean diet, omega-3 fatty acids, and polyphenol-rich foods as modulators of the gut-immune axis. Finally, we identify knowledge gaps, propose a research agenda prioritizing interventions with the potential to reduce inflammation-driven morbidity and mortality in PWH, and provide practical nutritional recommendations for clinicians managing PWH. The proposed pathways linking gut dysbiosis, microbial translocation, microbiome-targeted interventions, and persistent inflammation in people living with HIV are summarized in **Figure 1**.

As a narrative review, this manuscript was not intended to provide a systematic evaluation of all available literature. Studies were selected based on their relevance to the gut-immune axis, persistent inflammation, and microbiome-targeted and dietary interventions in PWH, with priority given to randomized controlled trials, systematic reviews, meta-analyses, and seminal mechanistic studies.

## 2 Prebiotics, probiotics, and synbiotics

### 2.1 Rationale for microbiome-targeted interventions

Persistent gut dysbiosis in PWH, marked by reduced levels of SCFA producing bacteria and increased presence of pro-inflammatory taxa, provides a compelling rationale for microbiome-targeted interventions (14, 28). Probiotics (live microorganisms), prebiotics (non-digestible substrates), and synbiotics (combinations of both) have emerged as low-risk strategies to modulate gut microbiota composition and function.

### 2.2 Probiotic interventions

Multiple probiotic formulations have been evaluated in PWH, with variable but generally encouraging effects on markers of microbial translocation and systemic inflammation. Agents studied in clinical trials include *Saccharomyces boulardii*, *Lactobacillus rhamnosus* GG (LGG), *Lactobacillus casei* Shirota, and multi-strain *Lactobacillus/Bifidobacterium* combinations.

*Saccharomyces boulardii*: In a double-blind, placebo-controlled trial of 44 virologically suppressed PWH (half of whom were immunologic non-responders with CD4+ counts < 270 cells/μL), 12 weeks of *S. boulardii* supplementation significantly reduced LBP and IL-6 compared to placebo (21). These reductions persisted at 3 months post-treatment discontinuation, suggesting durable effects. Mechanistically, *S. boulardii* was associated with decreased abundance of Clostridiaceae species that correlated with systemic inflammation markers. The investigators observed that immunologic non-responders exhibited significantly higher baseline LBP, sCD14, and systemic inflammation than immunologic responders, correlating with enrichment of Lachnospiraceae and Proteobacteria (22).

*Lactobacillus rhamnosus* GG: A prospective interventional trial of 45 PWH (15 ART-naive, 30 on ART) receiving LGG (6 × 10^9^ colony-forming units [CFU] twice daily) for 8 weeks demonstrated a significant decrease in intestinal inflammation as assessed by fluorodeoxyglucose positron emission tomography/magnetic resonance imaging (FDG-PET/MRI) imaging (p = 0.006) (28). This local anti-inflammatory effect was accompanied by significant reductions in pro-inflammatory gut taxa, including Enterobacteriaceae and Erysipelotrichaceae. Notably, however, no significant changes were observed in markers of microbial translocation (sCD14), macrophage activation (soluble cluster of differentiation 163 [sCD163]) or inflammation (hsCRP, IL-6, TNF-α), suggesting that LGG may exert predominantly local rather than systemic effects (28).

Multi-strain *Lactobacillus/Bifidobacterium* formulations: The Probio-HIV trial evaluated a multi-strain probiotic in 20 ART-treated PWH and demonstrated significant reductions in immune activation markers on CD4+ T cells expressing cluster of differentiation 38 (CD38) and human leukocyte antigen–DR (HLA-DR), as well as decreased LBP and hsCRP to levels comparable to HIV-seronegative controls (20) though the open-label design limits causal inference. These results indicate that probiotic supplementation may mitigate the persistent T-cell activation that characterizes treated HIV infection.

*Lactobacillus casei* Shirota: In contrast to the above findings, a double-blind, placebo-controlled pilot study of 48 PWH with poor CD4+ recovery found no significant effect of *L. casei* Shirota on CD4+ counts, CD4/CD8 ratio, immune activation markers, sCD14 levels, or gut microbiome composition at 12 weeks (29). This result highlights the strain-specificity of probiotic effects and underscores the need for careful selection of probiotic formulations.

Systematic reviews and meta-analyses have sought to reconcile the heterogeneous probiotic literature in PWH. A 2024 meta-analysis of 18 studies (including both RCTs and pre-post studies) found that probiotic supplementation modestly improved CD4+ lymphocyte counts by 53 cells/mm³ (95% confidence interval [CI]: 22–85), with quantitatively greater effects among ART-naive compared to ART-experienced individuals (30). However, a separate meta-analysis of 16 RCTs found no significant difference between probiotics/prebiotics/synbiotics and placebo on CD4+ counts (weighted mean difference [WMD] = 3.86, 95% CI: −24.72 to 32.45, p = 0.791), highlighting the inconsistency of findings across trials, with significant CD4+ improvements observed only in studies assessed as having high risk of bias (31). Probiotics have also been associated with reduced incidence of AIDS-related diarrhea (risk ratio [RR] = 0.60, 95% CI: 0.44–0.82), particularly with supplementation duration exceeding 30 days (32).

### 2.3 Prebiotic interventions

Prebiotics offer a complementary approach to probiotics by enhancing endogenous SCFA production. The COPA trial randomized 57 ART-naive PWH to receive a prebiotic oligosaccharide mixture containing short chain galactooligosaccharides (scGOS)/long chain fructooligosaccharides (lcFOS)/pectin hydrolysate-derived acidic oligosaccharides (pAOS) daily at 15 or 30 g daily or placebo for 12 weeks (33). Prebiotic supplementation significantly improved gut microbiota composition, with increased Bifidobacteria and decreased pathogenic *Clostridium* species. Immunologically, the prebiotic group demonstrated reduced sCD14, decreased CD4+cluster of differentiation 25 (CD25)+ T-cell activation, and enhanced natural killer (NK) cell activity compared to placebo.

A separate study evaluated a prebiotic mixture (scGOS/lcFOS/glutamine) in 44 PWH across different treatment strata (viremic untreated, ART-treated immunologic responders, and ART-treated immunologic non-responders) (34). Six weeks of supplementation reduced HIV-associated dysbiosis, most notably in viremic untreated individuals, with increased abundance of both *Faecalibacterium* and *Lachnospira* that were strongly associated with greater butyrate production and lower levels of sCD14 and hsCRP. The investigators concluded that the bacterial butyrate synthesis pathway represents a practical therapeutic target, directly linking prebiotic-induced microbiome changes to immunologic benefits.

### 2.4 Synbiotic interventions

Synbiotics have shown promise in PWH. A pilot RCT of 20 ART-naive PWH found that 16 weeks of synbiotic supplementation significantly increased CD4+ T-cell counts (median +102 cells/μL, p = 0.05), reduced plasma bacterial DNA concentrations (p = 0.048), and decreased IL-6 levels (p = 0.016) (35).

The RECOVER trial, a more recent double-blind, placebo-controlled study, randomized 71 immunodiscordant PWH (CD4+ <500 cells/mm³ despite viral suppression) to placebo, probiotic (*Lactobacillus/Bifidobacterium* strains), or synbiotic (probiotic plus fiber-based prebiotic) for 6 months (19). Synbiotic, but not probiotic alone, was associated with a significant increase in CD4/CD8 ratio at 6 months (p = 0.03), higher CD4+ counts at 9 months (447 vs. 342 cells/mL, p = 0.03), and lower sCD14 values (p = 0.008) compared to placebo. When probiotic was detectable in feces, significant decreases in CRP and IL-6 were observed, suggesting that either successful colonization or greater adherence to the intervention may be necessary to achieve anti-inflammatory effects. These findings support the hypothesis that combining probiotics with prebiotic substrates enhances immunological benefits, potentially by providing the fermentable substrate necessary for SCFA production. However, the clinical significance of these changes remains to be established.

Meta-analytic evidence from non-HIV populations supports the anti-inflammatory effects of synbiotics. A 2025 meta-analysis of 90 RCTs (5, 207 participants) found that synbiotic supplementation significantly reduced CRP (standardized mean difference [SMD] = −0.51), hsCRP (SMD = −0.66), TNF-α (SMD = −0.70), IL-1β (SMD = −1.35), and IL-6 (SMD = −2.02) in adults, with effects that numerically exceeded those of prebiotics alone for most markers (36). These findings provide mechanistic plausibility for synbiotic benefits in PWH, though HIV-specific trials remain limited.

## 3 Fecal microbiota transplantation and dietary interventions

### 3.1 Fecal microbiota transplantation

FMT offers the most direct strategy for restoring gut microbial ecology. A double-blind, placebo-controlled pilot study randomized 30 PWH on ART with CD4/CD8 ratio <1 to weekly FMT capsules or placebo for 8 weeks (24). Donors were deliberately selected based on high *Faecalibacterium* abundance and butyrate production capacity. FMT was safe, well-tolerated, and linked to significant increases in alpha diversity and temporary engraftment of donor microbiota, including Lachnospiraceae and Ruminococcaceae families generally lower in PWH. In exploratory analyses, FMT significantly reduced intestinal fatty acid-binding protein (IFABP), a biomarker of intestinal epithelial damage that is known to remain elevated despite viral suppression in HIV and independently predict mortality and morbidity in PWH (24, 37, 38).

Subsequent proteomic profiling of this cohort demonstrated that FMT was associated with significant reductions in 45 circulating inflammatory proteins, with these effects lasting up to 16 weeks after the final FMT procedure (23). The investigators identified specific bacterial taxa within Ruminococcaceae, Succinivibrionaceae, Prevotellaceae, and *Clostridium* genus that correlated with changes in inflammatory markers, providing mechanistic insight into the bacterial drivers of inflammation in PWH. These findings position FMT as a promising intervention capable of modulating the inflammatory proteome, though larger trials with clinical endpoints are needed.

### 3.2 Dietary interventions

While probiotic, prebiotic, and synbiotic interventions directly target the gut microbiome, an upstream and potentially more sustainable approach involves dietary modification to reshape microbial ecology and reduce inflammation. The Mediterranean diet, omega-3 fatty acids, and polyphenol-rich foods have demonstrated immunomodulatory effects in non-HIV populations, yet their application in PWH remains largely unexplored. This section synthesizes evidence on dietary interventions as modulators of the gut-immune axis, with emphasis on mechanisms relevant to HIV-associated inflammation.

#### 3.2.1 The Mediterranean diet

The Mediterranean diet, characterized by high intake of fruits, vegetables, legumes, whole grains, nuts, olive oil, and fish, with moderate wine consumption and limited red meat, is recognized as one of the healthiest dietary patterns globally and is associated with reduced cardiovascular disease, metabolic syndrome, and all-cause mortality (27). Emerging evidence indicates that these benefits are partially mediated through favorable modulation of the gut microbiome and intestinal inflammatory pathways. In non-HIV populations, higher Mediterranean diet adherence has also been associated with greater alpha diversity and enrichment of *Prevotella* and other diet-responsive taxa (39). These findings are consistent with broader evidence positioning the Mediterranean diet as a microbiome-modulating dietary strategy (27). Evidence suggests this association extends to PWH, where high Mediterranean diet adherence was similarly linked to increased gut microbiota alpha diversity, though markers of gut barrier dysfunction and systemic immune activation were similar regardless of diet adherence (25).

The Genetic, Environmental, Microbial (GEM) Project evaluated dietary patterns and gut microbiome composition in 2, 289 healthy first-degree relatives of patients with Crohn’s disease (26). Unsupervised clustering identified a Mediterranean-like dietary pattern (dietary component 3 [DC3]) that was strongly associated with enrichment of fiber-fermenting bacteria, including *Ruminococcus* and *Faecalibacterium*, taxa commonly depleted in PWH. The Mediterranean-like dietary pattern was associated with reduced subclinical gut inflammation, as reflected by fecal calprotectin levels. Mediation analysis demonstrated that this anti-inflammatory effect was partially mediated by the microbiota. These findings establish a mechanistic link between dietary pattern, microbiome composition, and intestinal inflammation.

Systematic reviews have suggested that Mediterranean diet adherence is associated with beneficial changes in gut microbiota diversity and composition, with consistent increases in *Faecalibacterium*, *Prevotella*, and enrichment of *Bacteroides* in observational studies, all fiber-degrading taxa (40). However, a separate systematic review found no consistent evidence that the Mediterranean diet uniformly alters gut microbiota composition or metabolism, citing substantial methodological heterogeneity across studies as a major limitation (41). The PREDIMED-Plus trial, a randomized controlled trial of 362 overweight and obese adults with metabolic syndrome, found that gut microbiota shifts occurred in both energy-restricted and standard Mediterranean diet groups with changes in Lachnospiraceae NK4A136 positively associated with Mediterranean diet adherence (42). These microbiome changes were accompanied by improvements in glycemic control, triglycerides, and high-density lipoprotein (HDL) cholesterol.

The mechanisms underlying Mediterranean diet-microbiome interactions are multifactorial. The diet’s high fiber content provides substrate for SCFA production, while its unsaturated fatty acids (particularly from olive oil) and polyphenols exert prebiotic effects (27, 43). Bailey and Holscher (43) proposed that the Mediterranean diet reduces metabolic endotoxemia through modulation of gut microbiota composition, increasing SCFA production, and improving intestinal barrier function.

Despite the compelling rationale, evidence on Mediterranean diet effects in PWH remains limited. A recent study by Illanes-Álvarez et al. evaluated intestinal microbiota, barrier integrity, and inflammatory markers in 40 long-term virologically suppressed PWH with high CD4+ counts (25). PWH had significantly lower abundance of *A. muciniphila* and *Paraprevotella* bacteria implicated in gut barrier integrity, compared to healthy controls. High adherence to a Mediterranean diet was associated with greater alpha diversity of the intestinal microbiota, while markers of gut dysfunction and immune activation did not differ by level of adherence.

Two small trials have evaluated extra virgin olive oil (EVOO) supplementation in PWH. A single-arm study of daily EVOO (50 g) for 12 weeks in HIV-infected adults over 50 years of age found significant reductions in total cholesterol and increased intestinal microbiota alpha diversity in men, with a decrease in pro-inflammatory genera such as Dethiosulfovibrionaceae. Changes in low-density lipoprotein (LDL) cholesterol, triglycerides, and C-reactive protein were not statistically meaningful (44). A randomized crossover trial comparing EVOO (50 mL/day for 20 days) to refined olive oil demonstrated significantly lower hsCRP with EVOO among participants with high compliance (>90%) (45). These findings provide preliminary evidence that Mediterranean diet components may favorably modulate both inflammatory markers and the gut microbiome in PWH.

#### 3.2.2 Omega-3 fatty acids

Omega-3 polyunsaturated fatty acids (PUFAs), including eicosapentaenoic acid (EPA) and docosahexaenoic acid (DHA), modulate immune responses through several complementary pathways. Their incorporation into cellular membranes influences membrane fluidity and lipid raft organization, activation of peroxisome proliferator activated receptor gamma (PPAR-γ) inhibits NF-κB dependent inflammatory gene expression, and their metabolism into specialized pro-resolving mediators (SPMs) promotes the resolution of inflammation (46). These mechanisms are directly relevant to HIV-associated inflammation.

A 2020 meta-analysis of 9 RCTs (427 participants) evaluated omega-3 fatty acid supplementation in HIV-infected patients (47). Omega-3 supplementation significantly reduced C-reactive protein (SMD: −0.27, 95% CI: −0.48 to −0.07, p = 0.007) compared to placebo, though effects on IL-6 and TNF-α were not statistically significant in pooled analyses. Individual trials have demonstrated more robust effects on specific cytokines. Metkus et al. (48) randomized 48 HIV-infected patients with hypertriglyceridemia to 3.6 g/day omega-3-acid ethyl esters or placebo for 8 weeks and found significant reductions in both IL-6 (−39% vs. +29%, p = 0.006) and TNF-α in the omega-3 group, though baseline IL-6 and TNF-α were higher in the omega-3 group, potentially contributing to the observed reductions. Similarly, Coghill et al. (49) demonstrated that 12 weeks of omega-3 supplementation (3g/day) significantly decreased IL-6 concentrations in HIV and human herpesvirus 8 (HHV-8) co-infected Ugandans (−0.78 pg/ml vs. +3.2 pg/ml, p = 0.04).

Beyond systemic inflammation, omega-3 fatty acids may exert tissue-specific anti-inflammatory effects. Domingo et al. (50) conducted a randomized, double-blind, placebo-controlled trial of DHA supplementation (2 g/day) for 48 weeks in 84 ART-treated PWH with hypertriglyceridemia. DHA supplementation significantly reduced hsCRP and downregulated inflammatory gene expression in subcutaneous adipose tissue, including TNF-α and monocyte chemoattractant protein-1 (MCP-1). However, a placebo-controlled trial of omega-3 supplementation in virologically suppressed men living with HIV found no improvement in endothelial function despite a significant reduction in soluble tumor necrosis factor receptor-I (sTNFR-I) and a trend toward improved inflammatory markers, suggesting that anti-inflammatory effects may not uniformly translate to vascular benefit (51).

#### 3.2.3 Polyphenols and *A. muciniphila*

Polyphenols, a diverse class of bioactive compounds abundant in fruits, vegetables, tea, coffee, wine, interact reciprocally with the gut microbiota: they promote beneficial taxa and suppress pathogenic organisms, while microbial enzymes convert them into metabolites with enhanced bioavailability and anti-inflammatory properties (52, 53).

Meta-analytic evidence from non-HIV populations demonstrates that polyphenol supplementation significantly reduces pro-inflammatory markers. A 2024 meta-analysis of randomized controlled trials in overweight and obese individuals found that polyphenols decreased the abundance of Firmicutes, Proteobacteria, Firmicutes-to-Bacteroidetes ratio and LPS while increasing beneficial bacteria including *Bifidobacterium* and *A. muciniphila* (54). A 2026 meta-analysis of 32 RCTs reported that polyphenol interventions were associated with reduced IL-6 and increased anti-inflammatory interleukin-10 (IL-10) in overweight and obese adults, with subgroup analyses suggesting larger effects at higher daily doses (>400 mg) and when delivered as whole polyphenol-rich foods rather than purified extracts (55).

Polyphenols may modulate inflammatory pathways via suppression of NF-κB signaling, reduction of pro-inflammatory cytokine production, mitigation of oxidative stress (52), as well as promotion of SCFA-associated microbial metabolism through prebiotic effects on microbial communities (53). Importantly, polyphenols promote commensal bacteria including *Lactobacillus*, *Bifidobacterium*, and *A. muciniphila* while suppressing pathogenic organisms including *Escherichia coli*, *Clostridium perfringens*, and *Helicobacter pylori* (53). Dietary fiber and polyphenols may act synergistically to enhance microbial diversity, SCFA production, and gut barrier function (56).

In the context of HIV, polyphenol-rich interventions may be particularly relevant given the reported reductions in *A. muciniphila* observed in PWH. Ouyang et al. proposed that interventions with high polyphenol content may increase *A. muciniphila* abundance based largely on evidence from metabolic contexts, with potential relevance for reducing microbial translocation and inflammation in PWH (12).

*A. muciniphila*, a mucin degrading bacterium that inhabits the intestinal mucus layer, has been identified as a sentinel for gut barrier integrity and a potential therapeutic target in PWH (12). This bacterium accounts for approximately 1–4% of the total fecal microbiota and has been reported to be reduced in several HIV cohorts, with lower abundance correlating with markers of microbial translocation and systemic inflammation supporting its proposed role in gut barrier dysfunction (12, 57).

The mechanisms by which *A. muciniphila* exerts favorable effects are being increasingly characterized, though important translational gaps remain. The bacterium strengthened epithelial monolayer integrity *in vitro*, as demonstrated by increased transepithelial electrical resistance in co-culture models (58). Its outer membrane protein Amuc_1100 activates toll-like receptor 2 (TLR2)-associated signaling and induces immunomodulatory cytokine responses, including IL-10 (57, 59). In addition, *A. muciniphila* contributes to SCFA-associated metabolism directly and through cross-feeding interactions, supporting mucosal integrity and regulation of systemic inflammation (60).

Strategies associated with increased *A. muciniphila* abundance include polyphenol-rich foods, Mediterranean dietary patterns, and prebiotic interventions, although much of this evidence derives from broader metabolic and gut health contexts rather than HIV-specific trials (12, 60, 61). Nonetheless, the convergence of these interventions on *A. muciniphila* enrichment suggests that this bacterium represents a potential biomarker for gut-immune axis modulation in PWH.

A summary of the major dietary and microbiome-targeted interventions discussed in this review, including representative studies, proposed mechanisms, and key findings, is provided in **Table 1**.

**Table 1:** Dietary and microbiome-targeted interventions with potential relevance to people living with HIV.

Intervention	Representative studies	Proposed mechanism(s)	Key findings
Probiotics	20, 21, 28	Barrier integrity; immune modulation; reduced microbial translocation	Reduced microbial translocation and inflammatory biomarkers in some studies; strain-specific effects observed
Prebiotics	33, 34	SCFA production; butyrate generation; enrichment of beneficial taxa	Improved microbiota composition, enrichment of SCFA-producing taxa, and reduced markers of microbial translocation
Synbiotics	19, 35	Combined probiotic and prebiotic effects; enhanced SCFA production; immune regulation	Improved inflammatory biomarkers and indices of immune recovery
Fecal microbiota transplantation	23, 24	Donor engraftment; microbial diversity restoration; ecosystem remodeling	Increased microbial diversity, reduced intestinal epithelial damage, and altered inflammatory proteome
Mediterranean dietary pattern	25, 26^†^; 27^†^	Fiber; polyphenols; unsaturated fats; SCFA production	Greater microbial diversity and a more favorable microbial profile; evidence in PWH remains limited
Extra virgin olive oil	44, 45	Polyphenol-mediated effects; antioxidant activity; microbiota modulation	Improved inflammatory biomarkers and increased microbial diversity in small studies
Omega-3 fatty acids	47–50	Cytokine modulation	Reduced systemic inflammation, although biomarker findings remain inconsistent
Polyphenol-rich foods and supplementation	52^†^; 53^†^; 54^†^; 55^†^; 12	Enrichment of beneficial taxa (including *A. muciniphila)*; promotion of SCFA-associated metabolism	Favorable effects on microbial composition and inflammatory signaling; HIV-specific data remain limited

NF-κB, nuclear factor kappa B; PWH, people with HIV; SCFA, short-chain fatty acid.

^†^Study conducted in a non-HIV population.

## 4 Conclusions and future directions

The gut-immune axis has emerged as a central mediator of persistent inflammation in PWH, offering a compelling therapeutic target beyond antiretroviral therapy alone. This review has synthesized evidence demonstrating that HIV-associated gut dysbiosis is marked by reduced SCFA producing bacteria and increased pro-inflammatory taxa, and compromised epithelial barrier integrity drives microbial translocation and systemic immune activation that persist despite virologic suppression. Nutritional and microbiome-targeted interventions represent a promising approach to addressing this residual inflammatory burden.

### 4.1 Knowledge gaps

Despite the growing evidence base, the following knowledge gaps limit clinical translation:

#### 4.1.2 Lack of hard clinical endpoints

Most nutritional and microbiome intervention studies in PWH have relied on surrogate biomarkers (IL-6, hsCRP, sCD14, CD4/CD8 ratio) rather than clinical events. Although these biomarkers are associated with mortality and non-AIDS morbidity in observational studies, it remains unproven whether interventions that reduce these markers translate into reduced cardiovascular events, malignancy, or death. The REPRIEVE trial provides a model for how adequately powered, endpoint-driven trials in PWH can be designed to determine whether biomarker modulation ultimately yields meaningful clinical benefit.

#### 4.1.3 Heterogeneity in intervention design

Probiotic literature is characterized by marked heterogeneity in strains, doses, formulations, and intervention durations, limiting cross-study comparability and making it difficult to identify optimal interventions. Similarly, prebiotic and synbiotic trials have employed diverse oligosaccharide compositions and combination strategies without standardization. This variability complicates interpretation of efficacy and may partially explain inconsistent findings across studies. Future research should prioritize standardized intervention protocols, head-to-head comparisons of specific formulations and dose-finding studies to clarify which microbiome-targeted strategies are most effective.

#### 4.1.4 Undefined role of baseline microbiome composition

Individual responses to microbiome-targeted interventions likely depend in part on baseline microbial ecology, yet few, if any, trials in PWH have stratified participants by baseline microbiome composition or identified predictive biomarkers of response. As a result, it remains unclear which individuals are most likely to benefit from specific probiotic, synbiotic, dietary, or FMT-based strategies. Precision microbiome medicine, tailoring interventions to individual microbial and host profiles, remains aspirational but is increasingly feasible with advances in metagenomic sequencing, multi-omic profiling, and machine learning.

#### 4.1.5 Limited dietary intervention research

Despite strong mechanistic rationale, randomized controlled evidence evaluating Mediterranean-style dietary interventions in PWH remains limited. This represents a critical gap given the diet’s established cardiometabolic benefits in the general population and its potential to simultaneously influence multiple pathways relevant to HIV-associated inflammation.

#### 4.1.6 Incomplete understanding of the gut virome and mycobiome

Most microbiome research in HIV has focused predominantly on bacterial communities, leaving the roles of the gut virome (particularly bacteriophages) and mycobiome (fungal communities) comparatively underexplored. This represents an important knowledge gap, as evidence suggests that alterations in bacteriophage populations may influence bacterial ecology and exacerbate dysbiosis, while fungal dysbiosis may drive mucosal inflammation. A more complete understanding of HIV-associated gut dysfunction will likely require multi-kingdom approaches.

### 4.2 Future research directions

Based on the evidence reviewed, we propose the following research priorities:

#### 4.2.1 Mediterranean diet intervention trials in PWH

While an ongoing randomized study (VIHMET; NCT06757309) is evaluating effects on immune activation and inflammatory biomarkers, additional studies are necessary to confirm findings, incorporate microbiome endpoints, and assess long-term clinical outcomes. Such trials should be of sufficient duration (≥12 months) to detect clinically meaningful changes.

#### 4.2.2 Standardized synbiotic formulations

Given emerging evidence that synbiotics may provide greater benefit than probiotics alone, future research should focus on developing and testing standardized synbiotic formulations optimized for PWH, potentially combining *S. boulardii* or multi-strain *Lactobacillus/Bifidobacterium* with prebiotic oligosaccharides that enhance butyrate production.

#### 4.2.3 Larger FMT trials

Building on pilot data demonstrating FMT’s effects on the inflammatory proteome, larger trials are needed to evaluate FMT’s impact on mechanistic pathways, validated inflammatory and cardiometabolic biomarkers, and longer-term clinical outcomes in high-risk PWH. Rational donor selection based on microbiome signatures (high *Faecalibacterium*, butyrate production capacity) should be standardized.

#### 4.2.4 Multi-omics approaches

Future studies should integrate metagenomics, metabolomics, and proteomics to characterize the functional consequences of microbiome interventions. Identifying specific bacterial metabolites (SCFAs, secondary bile acids) and their downstream effects on immune signaling will enable more targeted therapeutic development.

#### 4.2.5 Precision microbiome interventions

At present, insufficient evidence exists to guide microbiome-targeted therapies based on individual patient characteristics in PWH. However, future precision approaches may incorporate baseline microbiome composition, host genetics, ART exposure, and immune status to identify individuals most likely to benefit from specific interventions. For example, depletion of SCFA-producing taxa may identify individuals most likely to respond to dietary, prebiotic, or synbiotic strategies aimed at enhancing butyrate production, whereas those with more profound dysbiosis may require alternative or combination approaches. Similarly, differences in ART regimen, degree of immune recovery, and comorbidity burden may influence both baseline microbiome composition and treatment responsiveness. Integration of metagenomics, metabolomics, and host immune profiling may ultimately facilitate personalized intervention selection in PWH. Therefore, future research should prioritize approaches that account for interindividual variability in microbial composition, host characteristics, and immune status. Machine learning applied to multi-omics data may further improve prediction of individual responses to microbiome-targeted therapies.

#### 4.2.6 Combination strategies

Given the multifactorial nature of HIV-associated inflammation, combination approaches integrating dietary modification, targeted probiotics/synbiotics, and potentially FMT may offer greater potential for efficacy than single-intervention strategies alone. Such combination strategies warrant evaluation in factorial trial designs powered to assess mechanistic pathways, inflammatory and cardiometabolic biomarkers, and longer-term inflammation-driven comorbidities, including cardiovascular events and non-AIDS morbidity.

#### 4.2.7 The gut virome and mycobiome

Emerging evidence on fecal virome transplantation (FVT) and antifungal strategies suggest that targeting non-bacterial components of the gut ecosystem may offer additional therapeutic benefit. Future research should characterize the contributions of bacteriophages and fungi to HIV-associated inflammation and evaluate interventions targeting these kingdoms.

### 4.3 Clinical implications

While awaiting definitive trial evidence, clinicians caring for PWH can implement evidence-based nutritional counseling as part of comprehensive HIV care. Current guidelines from the HIV Medicine Association recommend routine counseling on lifestyle modifications, including a healthier diet with increased dietary fiber (62). Based on the evidence reviewed, practical recommendations include:

#### 4.3.1 Increase dietary fiber intake sufficient to meet the Adequate Intake: 25 g/day for women and 38 g/day for men (or 14 g per 1, 000 kcal)

The Academy of Nutrition and Dietetics supports these sex-specific targets based on evidence showing protection against coronary heart disease (63). A 2024 *NEJM* review notes that fiber intakes of approximately 25–29 g/day are associated with risk reductions across multiple health outcomes (64). Emphasize whole grains, legumes, fruits, and vegetables to provide fermentable substrate.

#### 4.3.2 Encourage Mediterranean dietary patterns rich in fruits, vegetables, legumes, whole grains, olive oil, and fish

A 2025 meta-analysis of 33 RCTs (n=3, 476) in non-HIV populations demonstrated that the Mediterranean diet was associated with significant reductions in hs-CRP, IL-6, and interleukin-17 (IL-17) compared to control diets (65). Preliminary evidence in PWH suggests that a key Mediterranean diet component, extra virgin olive oil, may also confer anti-inflammatory benefits (45).

#### 4.3.3 Incorporate omega-3 fatty acids

Encourage fatty fish 2–3 times weekly as part of an overall anti-inflammatory dietary pattern. For PWH with hypertriglyceridemia, omega-3 supplementation (2.4–4 g/day total omega-3 fatty acids) may be considered in line with established lipid guidelines, with potential for ancillary anti-inflammatory benefits supported by HIV-specific RCTs and meta-analytic evidence suggesting significant reductions in CRP, alongside more variable effects on IL-6 and TNF-α (47–49, 66, 67).

#### 4.3.4 Emphasize polyphenol-rich foods

Emphasize polyphenol-rich foods, particularly proanthocyanidin (PAC)-rich berries and grapes as well as epigallocatechin-3-gallate (EGCG)-containing tea, which may support *A. muciniphila* and broader favorable microbiome remodeling. Preclinical and mechanistic studies suggest that grape polyphenols, PACs, and EGCG may enhance *A. muciniphila* through multiple pathways, including PAC-mediated iron acquisition (xenosiderophore-like activity), modulation of gastrointestinal redox status, and substrate dependent co-metabolism (68–71). In a high-fat diet murine model, grape polyphenols substantially increased *A. muciniphila* abundance, reduced the Firmicutes-to-Bacteroidetes ratio and decreased intestinal expression of inflammatory markers (TNF-α, IL-6) (69).

#### 4.3.5 Consider selected probiotic or synbiotic interventions on an individual basis

Specific strain- and formulation-dependent probiotic or synbiotic interventions, including *S. boulardii* and selected multi-strain formulations, may modestly reduce markers of microbial translocation and systemic inflammation in some PWH, although findings remain inconsistent and broader clinical benefits remain unproven.

#### 4.3.6 Limit saturated fat and added sugar

Diets high in saturated fat have been associated with reduced microbial diversity, increased Firmicutes-to-Bacteroidetes ratio, and systemic endotoxemia through increased intestinal permeability (72). A 2021 study published in *Gut* reported that processed and animal-derived foods were consistently linked to pro-inflammatory microbial features and enrichment of endotoxin synthesis pathways, while plant foods and fish were positively associated with SCFA-producing commensals (73). Added sugars may contribute to depletion of SCFA-producing bacteria, impaired gut barrier integrity, and translocation of endotoxin across the gut barrier (74). In murine models, high-fructose and high-fat diets have been linked to chronic intestinal inflammation through microbiota-mediated mechanisms, including increased pro-inflammatory cytokines (IL-1β, IL-6) and decreased 5-hydroxytryptamine (75).

These recommendations carry minimal risk and align with general cardiovascular preventionstrategies, making them appropriate for routine clinical practice even in the absence of HIV-specific endpoint trials.

### 4.4 Concluding remarks

Nutritional and microbiome-targeted interventions including prebiotics, probiotics, synbiotics, dietary modification, and FMT, offer promising adjunctive strategies to complement effective ART. The convergence of these interventions on common mechanisms (SCFA production, favorable microbiome remodeling, gut barrier restoration) suggests that the gut microbiome may serve as a central therapeutic target. However, the field remains in its early stages, with gaps in our understanding of optimal intervention type, timing, durability, individual predictors of response, strategies for personalization, and whether reductions in inflammatory biomarkers translate into improved clinical outcomes. Addressing these gaps through rigorously designed clinical trials will be essential to realize the potential of nutritional immunomodulation in HIV care.
